# Comparison of nursing students’ performance of cardiopulmonary resuscitation between 1 semester and 3 semesters of manikin simulations in the Czech Republic: a non-randomized controlled study

**DOI:** 10.3352/jeehp.2023.20.9

**Published:** 2023-03-31

**Authors:** Vera Spatenkova, Iveta Zvercova, Zdenek Jindrisek, Ivana Veverkova, Eduard Kuriscak

**Affiliations:** 1 Faculty of Health Studies, Technical University of Liberec, Liberec, Czech Republic; 2 Neurointensive Care Unit, Neurocenter, Regional Hospital, Liberec, Czech Republic; 3 Institute of Physiology, First Medical Faculty, Charles University Prague, Prague, Czech Republic; Hallym University, Korea

**Keywords:** Cardiopulmonary resuscitation, Manikins, Nursing education, Nursing students

## Abstract

**Purpose:**

This study aimed to assess the effect of simulation teaching in critical care courses in a nursing study program on the quality of chest compressions of cardiopulmonary resuscitation (CPR).

**Methods:**

An observational cross-sectional study was conducted at the Faculty of Health Studies at the Technical University of Liberec. The success rate of CPR was tested in exams comparing 2 groups of students, totaling 66 different individuals, who completed half a year (group 1: intermediate exam with model simulation) or 1.5 years (group 2: final theoretical critical care exam with model simulation) of undergraduate nursing critical care education taught completely with a Laerdal SimMan 3G simulator. The quality of CPR was evaluated according to 4 components: compression depth, compression rate, time of correct frequency, and time of correct chest release.

**Results:**

Compression depth was significantly higher in group 2 than in group 1 (P=0.016). There were no significant differences in the compression rate (P=0.210), time of correct frequency (P=0.586), or time of correct chest release (P=0.514).

**Conclusion:**

Nursing students who completed the final critical care exam showed an improvement in compression depth during CPR after 2 additional semesters of critical care teaching compared to those who completed the intermediate exam. The above results indicate that regularly scheduled CPR training is necessary during critical care education for nursing students.

## Introduction

### Background/rationale

Simulation teaching is a modern education and teaching strategy that allows, through instructional scenarios, the creation of a reality in which students interact and where teaching takes place in conjunction with practical demonstrations on simulators that facilitate understanding and memorization [1]. It is a method of practical teaching through experience, sometimes also described as experiential learning, which can significantly aid learning and comprehension and improve critical thinking and self-directed learning [2].

The simulation-based teaching approach stresses the training of practical skills [3], which is especially important in acute medical states [4], life-threatening conditions, and emergency procedures such as cardiopulmonary resuscitation (CPR) [5]. It is generally assumed that students who participate in the simulation learning process [6] are better prepared to solve real critical care (CC) situations [7].

### Objectives

This study aimed to compare nursing students’ performance of CPR between students completing 1 semester of CC simulation teaching and students completing 3 semesters of teaching. Four components of CPR, including the compression depth, compression rate, time of correct frequency, and time of correct chest release, were assessed. CPR performance was evaluated during the end-of-semester examinations. The results may be helpful for evaluating teaching activities and reassessing the optimal ratio between CPR quality and teaching hours in didactic plans. Our hypothesis was that students who had completed a 3-semester CC course would show better CPR performance than students who had completed a 1-semester course.

## Methods

### Ethics statement

Ethical approval and consent to participate were not required according to the national law Act No. 111/1998 Coll., On Universities, which governs the Technical University of Liberec, because this study presents the results of a regular curriculum evaluation. No sensitive information was included in the evaluation process.

### Study design

This was a non-randomized controlled study.

### Participants

**Fig. 1.**
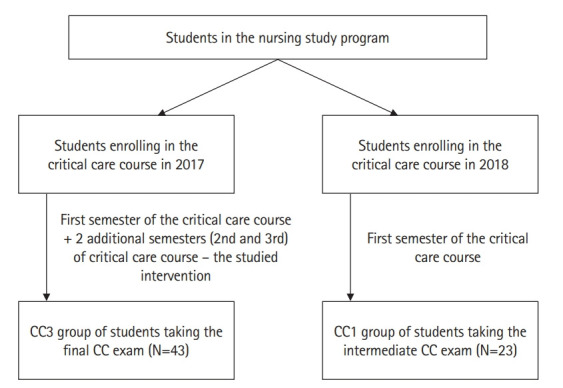
Flow diagram of the allocation of participants. CC, critical care; CC1, end-of-semester exam after the first semester of CC; CC3, end-of-semester exam after the third semester of CC.

In total, 66 students were involved in this study, forming 2 independent groups: the CC1 group of 23 individuals who completed the first semester of the CC course, and the CC3 group of another 43 individuals who completed 3 semesters of the CC education (first semester of the CC course plus 2 additional semesters of the CC course (Fig. 1). All students who took the CC1 and CC3 exams were included in the study. Fig. 1 shows the flow diagram of participants.

### Intervention

This study was conducted at the Faculty of Health Studies at the Technical University of Liberec, which has an accredited 3-year bachelor’s degree program for nurses. The performance of CPR was evaluated during January and February 2019, comparing 2 groups of students of CC education who had completed either 1 (group CC1) or 3 (group CC3) semesters of undergraduate nursing CC education. CC education is a mandatory part of the nursing degree and is taught over 3 semesters: throughout the entire second year, and during the first semester of the third (final) study year (CC1 takes place in the winter semester, CC2 in the summer semester, and CC3 in the winter semester). Since 2017, CC has been taught entirely in the form of simulation teaching on a patient manikin, with lectures and practice using solely this approach. In each semester, the CC lessons took place in blocks lasting 6 weeks, comprising six 90-minute-long lectures presented by an experienced CC physician who demonstrated various functionalities, feasible manipulations, and basic CC medical interventions on an adult manikin model (SimMan 3G, Laerdal Medical). In these lectures, students were close enough to the simulator to observe, touch, and explore various functions and simulated components (e.g., breathing movements and sounds, presence of the pulse on defined body parts, signal characteristics on bedside monitors, etc.), and to follow the progression of scenarios progressing over time according to the simulated or played script. After each lecture, students were split into 2 groups, each having a 45-minute session of practical training led by 2 non-physician teachers (master of science in CC plus bachelor of science in rescue service, both supported by 3 technicians setting up various real-time components of the patient simulator), who instructed students to work together as an efficient CC team, encountering a manikin patient model by solving various CC scenarios and exploring its behavior and functionality. This is, in essence, the description of the educational intervention analyzed in this study—namely, 2 additional semesters of CC teaching with lectures and practical sessions taught entirely on simulators (covering topics seen in the first column of Supplement 1) during which students were trained on CPR.

### Assignment method

Out of 66 students, 23 students who completed the first-semester CC course comprised the CC1 group and 43 other students who completed all 3 semesters of the CC course constituted the CC3 group. The assignment corresponds with the enrolment of students into the nursing study program starting in the academic year 2016/17 (CC3 group) and in 2017/18 (CC1 group).

### Blinding (masking)

There was no blinding of the intervention to participants.

### Outcome variables

The variables were CPR quality components, as follows: first, compression depth (50 to 60 mm was expected as the correct value); second, compression rate (the number of chest compressions in 1 minute; the expected correct frequency was 100 to 120 compressions per minute); third, the time of correct frequency (the percentage of time throughout the whole CPR session when the frequency was within 100–120 compressions/min; a percentage of 95%–100% was considered successful); and fourth, the time of correct chest release (the percentage of time throughout the whole CPR session when the chest was being correctly released; 95%–100% was considered successful.

### Data sources/measurement

All 4 CPR components were recorded during the CC-end-of-semester examinations, either at the end of the first-semester CC course (CC1 exam) or at the end of all 3 semesters of the CC course (CC3 exam). The CC1 exam consisted of 10 didactic standardized scenarios (Supplement 2) focusing on various static situations requiring immediate commencement of CPR upon arrival of students to the simulator. The CC3 exam comprised another standardized 10 CC situations that dynamically evolved over time (Supplement 1), requiring students to decide when CPR should start. The exam scenarios were the same scenarios that our students trained with over the year. Each student was supposed to review each scenario at least one time during his or her practice. All scenarios were of the advanced life support type (procedures extending basic live support, mainly involving cardiac monitoring by electrocardiography, airway management, and intravenous catheters for drug and fluid delivery). On average, the scenario lasted 10–15 minutes until students finished all tasks or it was terminated by the raters.

The success rate in all 4 mentioned CPR components was compared between the 2 groups, testing the effect of 2 more semesters on studied components. Raw response data of nursing students is available at Dataset 1.

### Bias

No known selection biases were identified.

### Study size

A priori sample size calculation was not possible because we had no reliable estimate for each component of the quality of CPR (compression depth, compression rate, time of correct frequency, and time of correct chest release). Therefore, we performed a *post hoc* power estimation of our data. Based on our results (Table 1), the *post hoc* power analysis indicated our study had 72.07%, 15.99%, 7.72%, and 13.49% power for each variable (compression depth, compression rate, time of correct frequency, and time of correct chest release, respectively) with an α of 0.05. The *post hoc* power analysis was conducted using G*Power software ver. 3.1.9.7 (Heinrich-Heine-Universität Düsseldorf).

### Unit of analysis

The unit of analysis was the same as the unit of assignment.

### Statistical methods

A statistical analysis was performed using Excel (Microsoft Corp.) and MathCracker Solvers Statistics (https://mathcracker.com/statistics-calculators-online). For the compression depth and compression rate, the consistency of variance was verified first, and then the 2-sample t-test was used. Because the time of correct frequency and time of correct chest release were calculated as percentages of the duration of the whole CPR session, making it very likely that they would not have a normal distribution, the Wilcoxon rank-sum test was used.

Understanding that the proper quality of resuscitation is of the utmost importance, and taking into account whether all evaluated CPR components were performed correctly at the same time by students, we arranged the data into pivot tables and calculated the relative frequencies of students who had tested components within the correct range. Since the numbers of students in the CC1 and CC3 groups differed, the relative success rates for each tested component were calculated and compared. The relative frequency is the percentage of students who correctly executed a given parameter among all students in the group. The Pearson chi-square test was used to test whether the relative success rate in the CC3 group was higher than in the CC1. For successful CPR, it is also important that the above-mentioned CPR components are kept correct simultaneously. Therefore, using the same test, we analyzed the proportions of students keeping 4, 3, 2, 1, and 0 components correctly (Table 2). Each field in Tables 2 and 3 contains the following 3 values: the observed relative frequency (cell totals in %), the expected relative frequency (cell totals in % that would match the null hypothesis stating that there would be no difference between the CC1 and CC3 groups - values in round brackets), and the calculated values of the chi-square statistic - values in square brackets. Data of statistical results is available at Dataset 2.

## Results

A comparison of the 4 measured components between the groups CC1 and CC3 can be seen in Table 1.

Table 4 shows the results of testing whether the compression depth exceeded 50 mm (the null hypothesis was set up for a depth ≥50 mm using the one-tailed single-sample t-test), indicating that the recommended depth of 50 mm was not reached in either group (for CC1, the t-value was -9.11, P<0.001; for CC3, the t-value was -5.44, P<0001).

Although the compression rates between the CC1 and CC3 groups did not differ significantly (Table 1), we were interested in whether they were within the recommended range of 100–120/min (Table 4). Two single-sample one-tailed t-tests, analyzing each CC group, showed that the compression rates in the CC1 and CC3 groups fell within the recommended range.

Tables 2 and 3 list the values of the chi-square test for the proportions of students successfully maintaining the given components of CPR within the recommended range. Table 3 shows the relative success rates for these components and demonstrates that the CC3 group had a higher success rate for compression depth than the CC1 group, but a lower rate of time of correct chest release. The success rate for the compression rate and the time of correct frequency did not differ significantly between the CC1 and CC3 groups.

For the proper quality of CPR chest compressions, it is also important that all measured CPR components are performed correctly at the same time. In Table 2, we can see the chi-square statistics for maintaining 4, 3, 2, 1, and 0 components simultaneously within the recommended range. For 4 successfully executed components, the chi-square statistic was 0.66, for 3 successfully executed components 8.26, for 2 it was 0.44, for 1 it was 3.50, and for 0 successfully executed components it was 1.08 (values smaller than 3.84 indicate no significant difference between CC1 and CC3 at a significance level of P<0.05). Thus, the students in the CC3 group were better at simultaneously maintaining 3 components than those in the CC1 group, but there were no significant differences in maintaining 4, 2, 1, and 0 components correctly.

## Discussion

### Key results

There was a statistically significant difference only in compression depth between these 2 groups, although we expected a much higher success rate in the CC3 group after 2 semesters of our CC teaching.

The success rate of maintaining 3 out of the 4 CPR components correctly was higher in the CC3 students than in the CC1 students (28% versus 13%); however, there were no significant differences in maintaining 4, 2, 1, or 0 components correctly. The proportions of students performing individual CPR components correctly at the same time are seen in Table 3, and the proportions of students performing 4, 3, 2, 1, or 0 components correctly are seen in Table 2.

### Interpretation

Although our study showed small improvements in some components of CPR after 2 semesters of teaching, this should not necessarily be interpreted as a reproach of our CC teaching. The results from this study should serve as important feedback and a reminder for us to focus on CPR training with unrelenting educational supervision, continuously focused on rehearsing those hard skills, regardless of how well soft skills (communication, time management, assertiveness, situational awareness, problem-solving, leadership, decision-making, etc.) are adopted or learned by our students. Moreover, teachers should ponder how to effectively use the students’ ability to solve complex situations or advanced scenarios in a synergistic way with their ability to execute CPR properly. For us teachers, this means that our supervision must be well balanced between emphasizing the understanding of CC situations simulated by advanced life support scenarios and rehearsing hard skills like CPR that can be simply mastered by repeated training.

### Comparison with previous studies

Similar studies focusing on the evaluation of acquired CPR skills by comparing teaching methods [5,8] or training frequency [9], have demonstrated the intricate manner of complexly evaluating the quality of CPR and its association with the teaching method and the number of student-hours. Those studies discussed the importance of defining an optimal teaching approach in regard to expected or required skills and knowledge and showed that the acquired CPR competency varied based on the type of manikin used, time spent on training and its frequency, and other factors such as receiving real-time visual feedback for instance.

### Limitations

We examined various model situations or scenarios taught in our CC courses, which could have made the CPR performance in our CC exams less uniform and homogenous. Our sample size was relatively small, which also reduced the power for the study outcomes. Therefore, the negative conclusions we made should be interpreted carefully and generalized cautiously.

### Generalizability

The presented results could have been influenced by other factors related to the characteristics of our students. Furthermore, not all hard skills related to CPR performance were tested on SimMan during resuscitation. These facts should be taken into account when generalizing our results regarding other teaching programs and different students.

### Suggestions

Based on our discussion of factors affecting CPR performance, we would like to admit that the methodology of evaluating the effect of soft skills on CPR performance is yet not fully elaborated [10]; however, it is becoming increasingly obvious that such skills must be incorporated [11] in any relevant analysis dealing with the complex issue of CPR quality.

### Conclusion

Although our study showed an improvement in CPR chest compression components after 2 semesters of a CC course taught solely on simulators, the improvement was not as pronounced as we expected. Our results suggest our educational supervision should be focused on more regular rehearsal of routine CPR, irrespectively of students’ progress in adopting and obtaining other skills and knowledge in their CC education.

## Figures and Tables

**Table 1.** Comparison of 4 measured components between the CC1 and CC3 groups

Parameter	CC 1 (N=23)	CC 3 (N=43)	P-value
Compression depth (mm)	40±5	44±7	0.016
Compression rate (min^-1^)	110±12	113±10	0.210
Time of correct frequency (%)	58±32	62±30	0.586
Time of correct chest release (%)	95±12	92±16	0.514

Values are presented as mean±standard deviation, unless otherwise stated. Only the compression depth increased significantly over the 2 semesters of CC courses. CC, critical care; CC1, end-of-semester exam after the first semester of CC; CC3, end-of-semester exam after the third semester of CC.

**Table 2.** The relative frequency (first value in %) of students in the CC1 and CC3 groups who executed 4, 3, 2, 1, or 0 parameters correctly during CPR (each number expresses an arbitrary combination of 4 tested CPR components)

Student group	No. of successfully executed components					Row total
	4	3	2	1	0	
CC1	4% (3.0%) [0.33]	9% (17.5%) [4.13]	43% (40.0%) [0.22]	35% (28.0%) [1.75]	9% (11.5%) [0.54]	100%
CC3	2% (3.0%) [0.33]	26% (17.5%) [4.13]	37% (40.0%) [0.22]	21% (28.0%) [1.75]	14% (11.5%) [0.54]	100%
Column total	6% [0.66]	35% [8.26]^a)^	80% [0.44]	56% [3.50]	23% [1.08]	200% (grand total)

Values in ( ) are the expected cell totals in % if the null hypothesis was valid (normalized to match % values in “row totals”), the numbers in [ ] are chi-square statistic summands, and their column-wise sums are the resulting values of the chi-square statistic confirming or rejecting the null hypothesis. CC, critical care; CC1, end-of-semester exam after the first semester of CC; CC3, end-of-semester exam after the third semester of CC; CPR, cardiopulmonary resuscitation.

a) Difference between the CC1 and CC3 groups at P<0.05 if the chi-square values are larger than 3.84.

**Table 3.** The relative frequency (% of the total number of students in the CC1 or the CC3 group) viewed as relative success rates in the tested components that were performed correctly by the given groups of students

Parameter	Compression depth	Compression rate	Time of correct frequency	Time of correct chest release	Row total
Correct value	50–60 mm	100–120/min	95%–100%	95%–100%	
CC1	4% (12.81%) [6.06]	57% (57.87%) [0.01]	13% (16.13%) [0.61]	83% (70.20%) [2.33]	157%
CC3	23% (14.19%) [5.46]	65% (64.13%) [0.01]	21 % (17.87%) [0.55]	65% (77.80%) [2.11]	174%
Column total	27% [11.52]^a)^	122% [0.02]	34% [1.16]	148% [4.44]^a)^	331% (grand total)

Values in ( ) are the expected cell totals in % if the null hypothesis was valid (normalized to match % values in “row totals”), the numbers in [ ] are chi-square statistic summands, and their column-wise sums are the resulting values of the chi-square statistic confirming or rejecting the null hypothesis. CC, critical care; CC1, end-of-semester exam after the first semester of CC; CC3, end-of-semester exam after the third semester of CC.

a) Difference between the CC1 and CC3 groups at P<0.05 if the chi-square values are larger than 3.84.

**Table 4.** Comparison of measured components with the 2015 guidelines for resuscitation

Parameter	Null hypothesis	Correct value according to guidelines	CC 1		CC 3	
			Mean±SD	P-value	Mean±SD	P-value
Compression depth	µ ≥50 mm	50 mm	40±5	<0.001	44±7	<0.001
Compression rate	µ ≤100/min	100–120/min	110±12	<0.003	113±10	<0.001
Compression rate	µ ≥120/min	100–120/min	110±12	<0.0001	113±10	<0.001

CC, critical care; CC1, end-of-semester exam after the first semester of CC; CC3, end-of-semester exam after the third semester of CC; SD, standard deviation.

## Footnotes

**Data availability**
Data files are available from Harvard Dataverse: https://doi.org/10.7910/DVN/W07OMI Dataset 1. Raw response data of nursing students.

Dataset 2. Data of statistical results.

## Supplementary materials

Supplementary files are available from Harvard Dataverse: https://doi.org/10.7910/DVN/W07OMI

Supplement 1. Model situations for the end-of-semester exam after the third semester of critical care.

Supplement 2. Model situations for the critical care 1 exam.

Supplement 3. Audio recording of the abstract.
