# Supplementary material for: Comparison of nursing students’ performance of cardiopulmonary resuscitation between 1 semester and 3 semesters of manikin simulations in the Czech Republic: a non-randomized controlled study
Source: J Educ Eval Health Prof. 2023 Mar 31;20:9. doi: 10.3352/jeehp.2023.20.9 (PMC10129870; doi:10.3352/jeehp.2023.20.9)
Supplement: Supplementary file 3 — Supplement 1. Model situations for the end-of-semester exam after the third semester of critical care. [file jeehp-20-09-suppl1.docx]

Supplement 1. Model situations for the end-of-semester exam after the third semester of critical care.

Table 2. Model situations for CC3 exam.

| 1. | Disorders of Consciousness. | Asystole |
| --- | --- | --- |
| 2. | Acute respiratory failure. | Asystole |
| 3. | Mechanical ventilation. | Asystole |
| 4. | Arrhythmia. | Ventricular fibrillation |
| 5. | Shock. | Asystole |
| 6. | Acute renal failure. | Ventricular fibrillation |
| 7. | Intoxication. | Asystole |
| 8. | Acidobasic disorders. | Asystole |
| 9. | Nutrition. | Ventricular fibrillation |
| 10. | Metabolism disorders. | Asystole |

CC – critical care; CC3 - end-of-semester exam closing the third semester of CC
