# Supplementary material for: Comparison of nursing students’ performance of cardiopulmonary resuscitation between 1 semester and 3 semesters of manikin simulations in the Czech Republic: a non-randomized controlled study
Source: J Educ Eval Health Prof. 2023 Mar 31;20:9. doi: 10.3352/jeehp.2023.20.9 (PMC10129870; doi:10.3352/jeehp.2023.20.9)
Supplement: Supplementary file 4 — Supplement 2. Model situations for the critical care 1 exam. [file jeehp-20-09-suppl2.docx]

**Supplementary materials**

Table 1. Model situations for CC1 exam.

| 1. | You are in a shopping centre and you go to the bathroom. There is someone locked inside WC, you can see the feet of the lying person under the door. The person does not react to your knocking on the door pand loud calling. | Ventricular fibrillation |
| --- | --- | --- |
| 2. | You are in the garden. Your neighbour tells you fearfully over the fence that her husband suddenly fell down when he was cutting the grass. | Asystole |
| 3. | You are sitting in a restaurant in a small town. From the back of the restaurant, which is out of your sight, you hear an urgent coughing. At that moment, panic and shouting occurs. You go to see what is going on. You find a woman lying. | Asystole |
| 4. | You are watching an ice hockey match in the local arena. The man sitting next to you (approx. 50-year-old) suddenly grabs his chest and immediately loses consciousness. | Ventricular fibrillation |
| 5. | You are going through the park, there is a big sports area nearby. You find a crumpled-up man, probably homeless. | Ventricular fibrillation |
| 6. | When you check the patients during your night shift at the inpatient department, you find an unconscious patient lying on the floor next to his bed. | Asystole |
| 7. | You are accompanying a female patient in the hospital to the Computer Tomography examination. The patient starts to complain of nausea and suddenly loses consciousness. | Ventricular fibrillation |
| 8. | In the gynaecologist´s waiting room, a young women in an advanced stage of pregnancy falls out of her seat, she has lost consciousness. | Ventricular fibrillation |
| 9. | Your friend and you go to visit her grandma. When you open the door, you find the grandma lying unconscious on the floor. Her clothes and mouth are bloody, you see a bucket with vomit and blood next to her bed. | Asystole |
| 10. | A person in a lake calls for help, sinks. | Asystole |

CC – critical care; CC3 - end-of-semester exam closing the third semester of CC
